# Supplementary material for: Iron Starvation Induces Ferricrocin Production and the Reductive Iron Acquisition System in the Chromoblastomycosis Agent Cladophialophora carrionii
Source: J Fungi (Basel). 2023 Jul 5;9(7):727. doi: 10.3390/jof9070727 (PMC10382037; doi:10.3390/jof9070727)
Supplement: Supplementary file 1 [file jof-09-00727-s001.zip › Tables.pdf]

Table S1. Orthology analysis of genes involved in high affinity iron acquisition in *C. carrionii***.**

Reference fungi	Sequence ID*	Protein name	Orthologs in *C. carrionii**	*E*-value*
**Siderophore biosynthesis**				
A. fumigatus	Afu2g07680	SidA	CLCR_04832 (SidA1)	$5e^{-174}$
			CLCR_02414 (SidA2)	$5e^{-55}$
	Afu3g03400	SidF	CLCR_02426	0.0
	Afu1g17200	SidC	CLCR_04490	0.0
	Afu3g03420	SidD	CLCR_02213	0.0
	Afu1g04450	SidL	CLCR_04002	0.0
	Afu1g17190	SidI	CLCR_02353	0.0
	Afu3g03410	SidH	CLCR_02613	$5e^{-80}$
**Siderophore transport**				
A. fumigatus	Afu3g03440	MirD	CLCR_02321	0.0
			CLCR_03365	0.0
			CLCR_06890	$5e^{-156}$
			CLCR_03414	$7e^{-136}$
			CLCR_01135	$8e^{-66}$
			CLCR_04614	$2e^{-50}$
	Afu3g03640	MirB	CLCR_03365	0.0
			CLCR_02321	0.0
			CLCR_06890	$2e^{-152}$
			CLCR_03414	$2e^{-137}$
			CLCR_01135	$4e^{-71}$
			CLCR_04614	$3e^{-48}$
	Afu7g04730	Sit2	CLCR_06890	$3e^{-153}$
			CLCR_02321	$5e^{-146}$
			CLCR_03365	$1e^{-123}$
			CLCR_03414	$2e^{-119}$
			CLCR_01135	$1e^{-56}$
			CLCR_04614	$9e^{-46}$
	Afu2g05730	MirC	CLCR_02321	$3e^{-104}$
			CLCR_06890	$8e^{-104}$
			CLCR_01135	$4e^{-101}$
			CLCR_03365	$1e^{-94}$
			CLCR_03414	$2e^{-84}$
			CLCR_04614	$4e^{-70}$
	Afu7g06060	Sit1	CLCR_01135	0.0
			CLCR_02321	$9e^{-72}$
			CLCR_06890	$4e^{-61}$
			CLCR_03414	$2e^{-60}$
			CLCR_03365	$4e^{-60}$
			CLCR_04614	$3e^{-51}$
A. nidulans	An8540	MirB	CLCR_03365	0.0
			CLCR_02321	0.0
			CLCR_06890	$4e^{-159}$
			CLCR_03414	$1e^{-133}$
			CLCR_01135	$3e^{-67}$
			CLCR_04614	$1e^{-50}$
	An7485	MirC	CLCR_02321	$6e^{-107}$
			CLCR_06890	$2e^{-102}$
			CLCR_01135	$8e^{-101}$

			CLCR_03365<br>CLCR_03414<br>CLCR_04614	$1e^{-94}$<br>$2e^{-84}$<br>$3e^{-69}$
	An7800	MirA	CLCR_02321<br>CLCR_03414<br>CLCR_06890<br>CLCR_01135<br>CLCR_03365<br>CLCR_04614	$7e^{-85}$<br>$5e^{-79}$<br>$5e^{-75}$<br>$6e^{-71}$<br>$2e^{-66}$<br>$5e^{-58}$
S. cerevisiae	YOL158C	Enb1p/Arn4p	CLCR_01135<br>CLCR_02321<br>CLCR_04614 CLCR_06890<br>CLCR_03365<br>CLCR_03414	$4e^{-102}$<br>$2e^{-53}$<br>$4e^{-49}$<br>$3e^{-48}$<br>$2e^{-45}$<br>$3e^{-37}$
	YHL047C	Taf1p/Arn2p	CLCR_01135<br>CLCR_06890<br>CLCR_02321<br>CLCR_04614<br>CLCR_03414<br>CLCR_03365	$7e^{-134}$<br>$1e^{-55}$<br>$2e^{-50}$<br>$7e^{-46}$<br>$2e^{-43}$<br>$4e^{-41}$
	YHL040C	Taf1p/Arn2p Arn1p	CLCR_01135<br>CLCR_02321<br>CLCR_06890<br>CLCR_03365<br>CLCR_04614<br>CLCR_03414	$6e^{-147}$<br>$7e^{-50}$<br>$3e^{-49}$<br>$4e^{-46}$<br>$4e^{-45}$<br>$7e^{-41}$
	YEL065W	Sit1p/Arn3p	CLCR_01135 CLCR_03414<br>CLCR_02321<br>CLCR_06890<br>CLCR_03365<br>CLCR_04614	$1e^{-167}$<br>$2e^{-52}$<br>$4e^{-47}$<br>$1e^{-46}$<br>$1e^{-44}$<br>$2e^{-43}$
C. neoformans	CNA07920	Sit1	CLCR_01135 CLCR_02321<br>CLCR_04614<br>CLCR_06890<br>CLCR_03365<br>CLCR_03414	0.0<br>$5e^{-64}$<br>$2e^{-61}$<br>$7e^{-59}$<br>$3e^{-55}$<br>$1e^{-51}$
**Reductive iron acquisition (RIA)**				
S. cerevisiae	YER145C	Ftr1	CLCR_07299<br>CLCR_03583	$4e^{-134}$<br>$4e^{-132}$
	YMR058W	Fet3	CLCR_03382<br>CLCR_06747	0.0<br>0.0
A. fumigatus	Afu5g03800	FtrA	CLCR_07299<br>CLCR_03583	$5e^{-161}$<br>$2e^{-141}$
	Afu5g03790	FetC	CLCR_06747<br>CLCR_03382	0.0<br>0.0
**Regulation of iron metabolism**				
A. fumigatus	Afu5g11260	SreA	CLCR_05818	$8e^{-68}$
	Afu5g03920	HapX	CLCR_01554	$2e^{-85}$

*According to FungiDB (https://fungidb.org/fungidb/app)

Table S2. Oligonucleotides used in qRT-PCR experiments.

Gene	Forward primer	Reverse primer
hapX	TGCACCTTGGAGACCCGCT	ACACTCTCTGGGTTCGAGCT
sreA	ACCTGAGCATACTTCCGGCA	CACTTGCTCCATCTTCGGGT
sidA1	AGTCGATCCTCCCACACAAG	CGGAGGAGTGCAGGACGC
sidI	GACATCATCATCAGGGGTGG	GCCTCCTCGCTGGTCTGC
ftrA1	GAAGCTGCGCAACCAGGTCT	GCGACTAGGGCGAACACAC
ftrA2	CTCGGTGCTGCTCTCGTTC	GGCCCAGACCGTAGAAGAC
act	TGGCAACGAACGATTCCGAG	CATAGTAGTACCACCAGACATG
