# Supplementary figures and images for: Iron Starvation Induces Ferricrocin Production and the Reductive Iron Acquisition System in the Chromoblastomycosis Agent Cladophialophora carrionii
Source: J Fungi (Basel). 2023 Jul 5;9(7):727. doi: 10.3390/jof9070727 (PMC10382037; doi:10.3390/jof9070727)

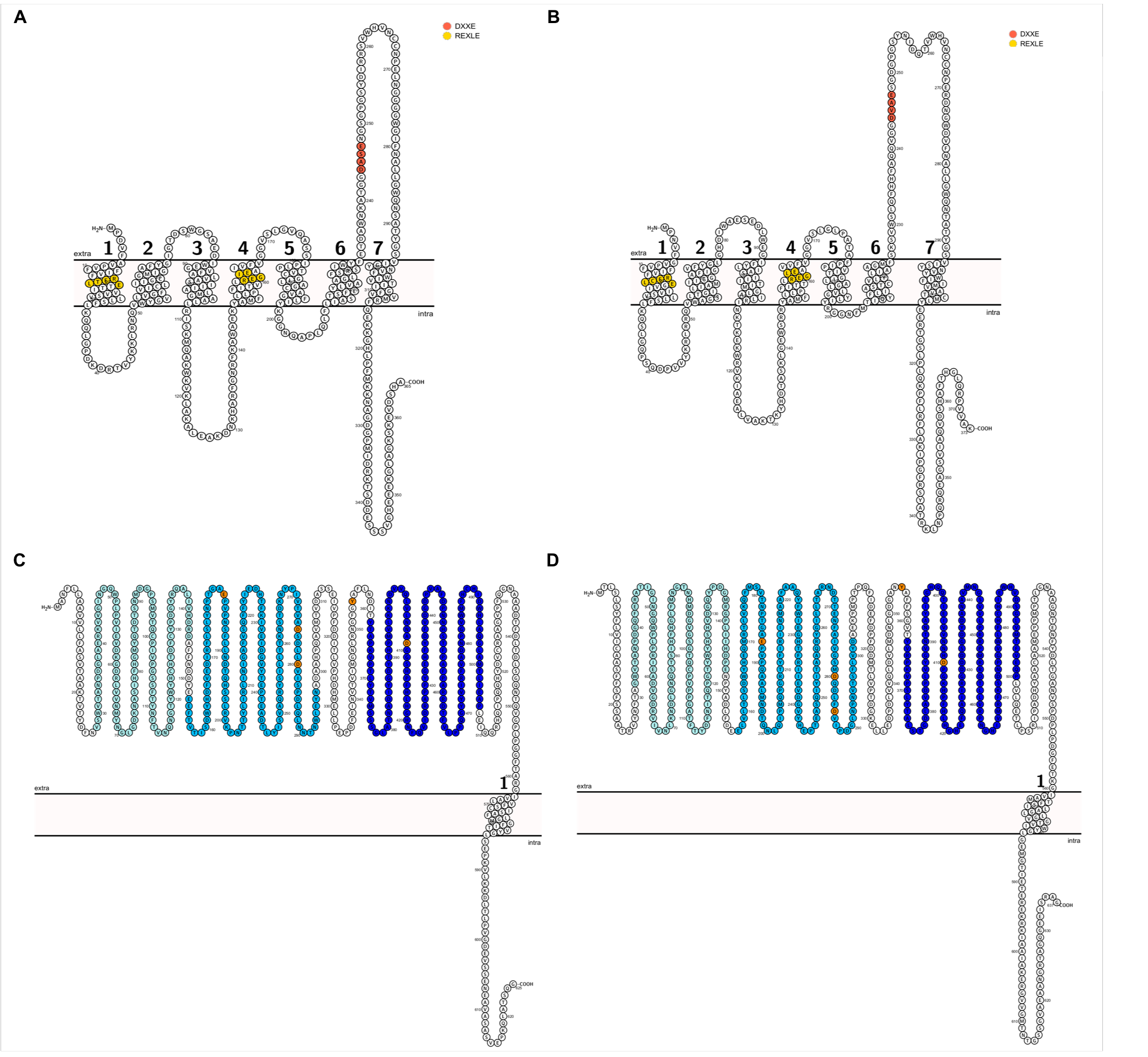

Supplement: Supplementary file 1 [file jof-09-00727-s001.zip › Figure S1.tiff]

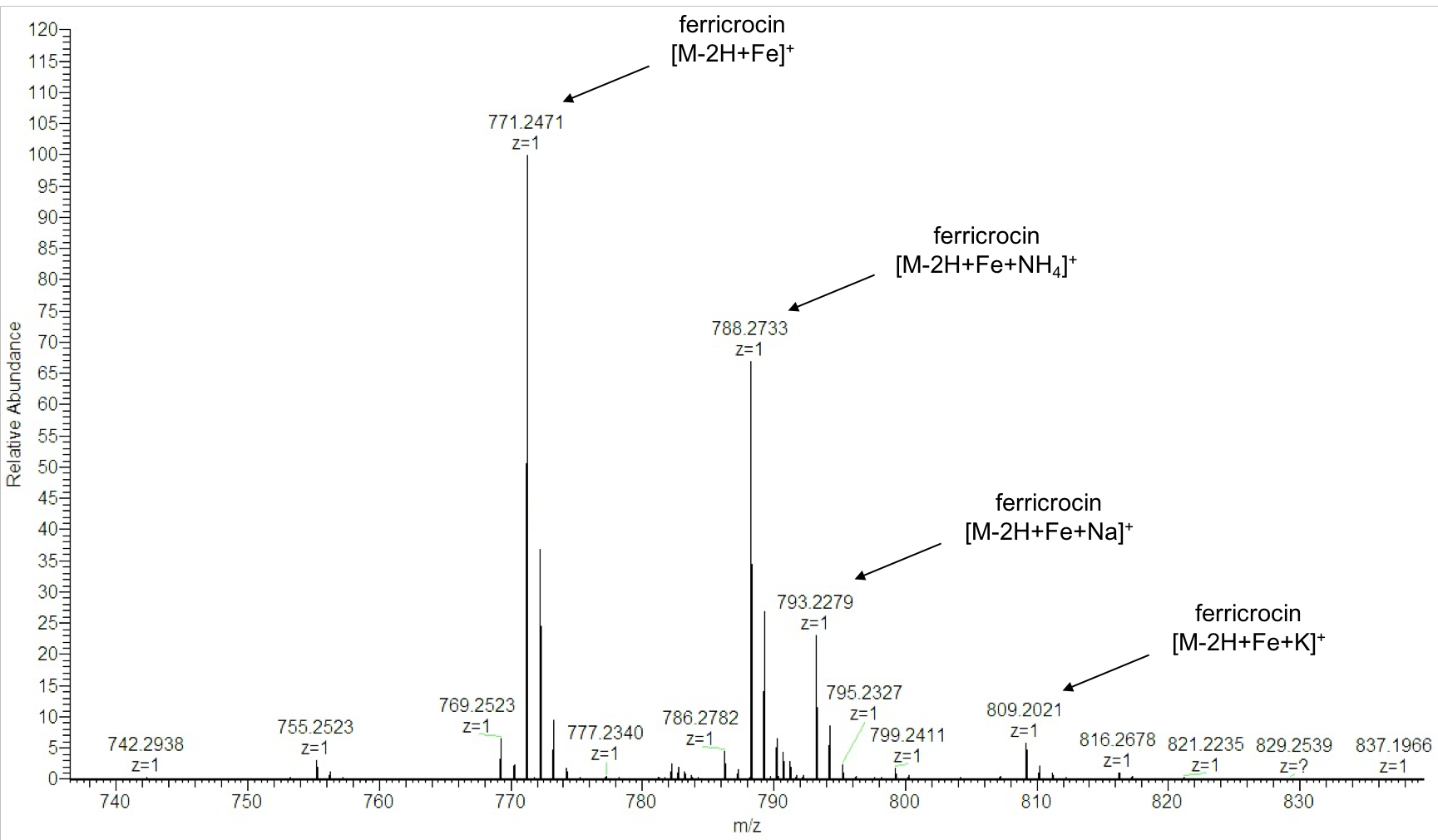

Supplement: Supplementary file 1 [file jof-09-00727-s001.zip › Figure S2.tiff]
